# Supplementary material for: Impact of body mass index on surgical and oncological outcomes after Hyperthermic Intraperitoneal Chemotherapy (HIPEC)
Source: PLoS One. 2025 Jul 7;20(7):e0325941. doi: 10.1371/journal.pone.0325941 (PMC12233287; doi:10.1371/journal.pone.0325941)
Supplement: Attachment — Submitted filename: Response to Reviewers.docx [file pone.0325941.s002.docx]

Response to Reviewers

**Reviewer #1: Summary:**

1 : One major comment concerning the choice of etiologies. I think that the authors have to make a decision what to look at. If you are to only evaluate morbidity and postop mortality, I think it is reasonable to include all etiologies even though we know the dissection in PMP differs from gastric and colorectal. Nonetheless, a case can be made to include all CRS for a postop morbidity comparison. However, a postop survival comparison cannot be done with all these etiologies mixed in. For example in table 4, obviously gastric peritoneal metastases have a much worse survival than PMP or colorectal. The hazard ratio is high over 2 but due to small numbers doesn’t actually reach statistical significance. As such, etiologies are not included in the final multivariable analysis of survival, but obviously the histopathology will have a great impact on the multivariable model. I suggest either skipping the survival analyses completely or do a limited subgroup analysis removing all the small size etiologies, ie keep only colorectal, appendix, and PMP. Even if the univariate analysis is non-significant, the etiologies need to be included in the multivariable analysis anyways.

- Thank you for your comments on the diversity of etiologies included in our study. We recognize the complexities and inherent differences in survival outcomes among various types of peritoneal metastases, such as pseudomyxoma peritonei, gastric, and colorectal cancers.
- We treated the postoperative morbidity of all patients included in this study. For the survival analysis, as advised, we focused solely on carcinosis of colorectal origin. We did not analyze the survival of pseudomyxoma, none of which were registered.
- We agree, and have modified our results and add statistical analyzes( 30-Day Mobidity line 164-170, page 10; 90-Day Morbidity 183-188, page 12 and OS page 15)

2 : If you choose to focus solely on morbidity which I think is the cleanest option. It would be interesting to have a logistical regression model with grade 3+ yes/no endpoint or readmission within 90 days as endpoint. Clinically, the BMI and complication rates are mostly contemplated not so much survival. The clinically most important point is the surgical risk and complication rate.

- While we have retained the overall structure of our article, we have enhanced the sections on 30-day and 90-day morbidity. These sections now include more detailed analyses that better capture the relationship between BMI and significant postoperative complications (Cf response comment 1). A logistic regression according to the occurrence or non-occurrence of a complication higher than grade 3 is a suggestion. We have improved the paragraphs concerning postoperative morbidity, but without adding this point, however, as it is somewhat similar to our analysis according to BMI higher or lower than 25.

3 : I suggest testing different BMI cut-off as a sensitivity analysis. Do things change with higher BMI cut-off? Please provide a little table with Clavien-Dindo morbidity, Readmission 90D, and Reintervention 90D according to BMI <35 or 35+. This cut-off is usually used for when gastric bypass is offered. Perhaps the number of patients will be too small, but even if not including in the article, please provide it for review purposes. If the results are similar, you could just add a sentence in the results section mentioning that cut-off of BMI 35 did not change the outcomes.

- We conducted this analysis and included this results in the results (table 3 and 4 page 11 and 13). However, our study had limited statistical power for this specific analysis due to the small number of patients with a BMI ≥35.

4 : Table 4 – This is almost a completely meaningless multivariable analysis. It is full of confounding variables as well as time dependant variables. This just does not suffice. For example, all the aggressive or poor prognostic etiologies (gastric and advanced colorectal) receive neoadjuvant therapy while the very good prognostic ovary and PMP usually do not. Thus, neoadjuvant therapy looks to have a terrible hazard ratio which is clearly misguiding. In other words, the neoadjuvant variable is directly affected by and determined by the etiologi variable. You have repeat patients as well. This group is by definition a selected group from a selected group compared to those doing their first CRS+HIPEC. You have adjuvant therapy included which also is determined by etiologi and postop complications. Intraperitoneal chemotherapy is also completely etiologi dependent. Mitomycin C is the standard drug for PMP which has a super good prognosis which makes it misguided. Since etiologi is not apart of the multivariable analysis, these differences are not adjusted correctly. This multivariable model is just not feasible or methodologically sound. See comment 1. I am not sure a Cox model for overall survival including all etiologies is feasible. Besides the limited number of patients, the other variables such as neoadjuvant and adjuvant chemo is never offered low grade PMP. So when you have this situation with 0 patients in some of the subgroups, the multivariable analyses tend to fail. In principle, all the baseline variables chosen need to be possible to fall into the included subgroups. If this is not the case, the model becomes problematic.

- In the revised manuscript, we have refined our focus to primarily address outcomes related to colorectal etiology. This decision was based on the homogeneity and volume of data available, allowing for a more robust and accurate analysis. We have limited our scope to avoid the methodological challenges previously encountered with more varied etiologies like PMP, for which, as you noted, no patients were receiving neoadjuvant or adjuvant therapy in our cohort. This adjustment ensures that the variables included in our analysis are applicable and significant to the subgroup studied, enhancing the validity and reliability of our findings.
- We agree, and have modified our results and add statistical analyzes (line 220 -236 page 15-16 and table 6 and 7 + figure 1)

5 : Basically, the same reasoning for Table 5 as it is for Table 4. I would suggest discussing with a statistician. My suggestion would be to skip the Cox regression modelling for overall and recurrence free survival and focus on the postop morbidity (and readmission) instead.

- As mentioned in our response to the earlier comment to comment 4, we have consulted with a statistician and subsequently refined our focus primarily on colorectal etiology and not only on morbidity.

**Reviewer # 2 Summary:**

The study explores the influence of body mass index (BMI) on surgical and oncological outcomes in patients undergoing cytoreductive surgery with hyperthermic intraperitoneal chemotherapy (HIPEC) for peritoneal metastases. The relevance of this research lies in the rising rates of obesity and overweight in Europe and the need to understand better how BMI impacts survival and postoperative complications in this context of intensive treatment.
This is a retrospective observational study conducted at two HIPEC expert centers in France from 2017 to 2021. Patients are divided into two groups based on BMI (<25 and ≥25). The primary endpoints are 30-day morbidity, overall survival (OS), and recurrence-free survival (RFS). The statistical analysis uses a Cox model to adjust for confounding variables, including sex, neoadjuvant chemotherapy, and the HIPEC techniques used (open or closed).
The results show that:

- Overall survival is significantly higher for patients with BMI≥25. The "obesity paradox" is suggested to explain this paradoxical association where a higher BMI is linked to better survival.
- 30-day morbidity is similar between both groups, although the high-BMI group showed an increased rate of digestive fistulas and 90-day readmissions.
- Factors such as male sex, neoadjuvant chemotherapy, and the use of mitomycin C in HIPEC also influence survival.

The article highlights an inverse association between BMI and postoperative mortality, supporting the "obesity paradox." This phenomenon is intriguing but remains debated, particularly in oncology. However, the study presents certain limitations that complicate interpretation:

- Heterogeneity of patients: Patients have peritoneal metastases from various origins (colorectal, pseudomyxoma, ovarian, etc.), which could introduce bias. Survival outcomes may vary depending on cancer type, making it difficult to attribute a specific effect of BMI on survival.
- Retrospective design: The retrospective nature of the study limits the robustness of conclusions. Data are subject to selection and reporting bias, and analysis relies on the quality of medical records.
- Lack of data on other confounding factors: Although the study adjusts for some factors like sex and HIPEC technique, other variables (e.g., precise nutritional status, unreported comorbidities) might also affect the outcomes.

The study concludes that a BMI≥25 might be protective for the overall survival of patients undergoing HIPEC treatment, but it calls for further studies to confirm these findings and explore additional factors in the long term. It also highlights the need for a better understanding of the obesity paradox in surgical oncology.

The study presents a relevant analysis on a clinically important topic. However, methodological biases and limitations reduce the strength of the conclusions. A prospective design or finer stratification of patient groups by pathology could enhance the validity of the results.

- Thank you for your thorough review and insightful comments. We appreciate your recognition of the relevance of our study in the context of rising obesity rates in Europe and its potential impact on surgical outcomes in HIPEC procedures.
- We acknowledge the limitations highlighted, particularly the heterogeneity of the patient population and the retrospective nature of the study, which might affect the robustness of our conclusions. In response, we have clarified these points in the manuscript, emphasizing that while our findings support the 'obesity paradox', they indeed call for cautious interpretation.

**Reviewer # 3 Summary::**

This retrospective two-center study examined the impact of body mass index (BMI) on surgical and oncological outcomes in 144 patients who underwent complete cytoreductive surgery with HIPEC for peritoneal metastases between 2017 and 2021. Patients with BMI<25 showed significantly poorer overall survival compared to those with BMI≥25, although recurrence-free survival was similar between groups. While 30-day complication rates were comparable between groups, patients with BMI≥25 experienced more digestive fistulas and higher 90-day readmission rates. The study identified several significant predictors of outcomes, including BMI<25 and male sex as risk factors for poorer overall survival, while pseudomyxoma histology emerged as a protective factor. Overall, the study is interesting and the manuscript is well-written. Thus, I just have one minor concern.

1. May add the subgroup analysis according to BMI to validate the significant association between BMI and clinical outcomes of the patients.

- We have added a detailed subgroup analysis focused on different BMI categories (table 3 and 4 page 11 and 13). This analysis further investigates the association between BMI and clinical outcomes, strengthening the validity of our findings. We have included this analysis in the revised manuscript, offering clearer insights into how different BMI ranges affect surgical and oncological outcomes.

2.	Please define the comorbidities, such as cardiopathy, mini-stroke, renal failure, hepatopathy in the table 1.

- We have updated Table 1 to include precise definitions of the comorbidities mentioned, such as cardiopathy, mini-stroke, renal failure, and hepatopathy.
