# Supplementary material for: Impact of body mass index on surgical and oncological outcomes after Hyperthermic Intraperitoneal Chemotherapy (HIPEC)
Source: PLoS One. 2025 Jul 7;20(7):e0325941. doi: 10.1371/journal.pone.0325941 (PMC12233287; doi:10.1371/journal.pone.0325941)
Supplement: Attachment — Submitted filename: Response_to_Reviewers_auresp_2.docx [file pone.0325941.s003.docx]

Response to Reviewers

**Reviewer #3:** The authors had addressed well all my concern, so I have no more suggestion for this revision. Congratulation
🡺 Thank you very much for your encouraging feedback and for acknowledging the revisions made to our manuscript. We are grateful for your thorough review

**Reviewer #4:** I read with interest the manuscript “Impact of body mass index on surgical and oncological outcomes after Hyperthermic Intraperitoneal Chemotherapy (HIPEC)”. The paper faced on an interesting topic, the evaluation of BMI in patients treated with CRS-HIPEC as risk factor for postoperative outcome and oncological long term results. The text is clear and easy to follow. However, there are important drawbacks of this work.

1-The number of patients is too low for draw any reliable conclusion
🡺 Thank you for your comment regarding the sample size. We acknowledge this concern; however, our sample size aligns with those commonly seen in similar HIPEC studies, ensuring our study's conclusions remain robust and comparable within this field (Gurusamy et al., 2024).

2-The impact of BMI on morbidity and mortality seems null. This is quite surprising. How explain the authors this result?
🡺 Thank you for your comment on the unexpected null impact of BMI on morbidity and mortality. This may be due to the rigorous patient selection and specialized care protocols at our high-volume centers, which can mitigate typical BMI-related risks. Our findings highlight the potential for advanced surgical expertise and care standardization to overshadow the usual influences of BMI on surgical outcomes. Further research could explore this phenomenon across diverse clinical settings.

3-A higher incidence of intestinal fistula is reported. How do the authors explain this result?
🡺 Thank you for your comment on the incidence of intestinal fistulas in our study. The observed rate of fistulas, while notable, is within the expected range for major abdominal surgeries, as supported by literature showing a similar incidence of around 5.5% in such procedures (Wercka J, Cagol PP, Melo AL, Locks Gde F, Franzon O, Kruel NF. Epidemiology and outcome of patients with postoperative abdominal fistula. Rev Col Bras Cir. 2016 Mar-Apr;43(2):117-23). Our study's results reflect this established complication rate, underscoring the challenging nature of these surgeries and the inherent risks involved, particularly in complex cases requiring HIPEC.

4-OS is higher in obese patients. How do the authors explain this result?
🡺 Thank you for your comment on the higher OS in obese patients. This finding may align with the "obesity paradox," where obesity sometimes correlates with better survival outcomes in specific medical scenarios. This could suggest that metabolic reserves in obese patients offer some resilience during recovery from major surgeries like HIPEC. Further research would be beneficial to clarify this observation.

However, the main problem of the manuscript is the discussion where none of the results of the study have been discussed or interpreted. Moreover, the authors didn’t highlight the importance of their results. Why are the results important? How can it change the clinical practice or set the ground of future studies?
🡺 Thank you for your valuable feedback regarding the discussion section of our manuscript. We have carefully considered your comments and have expanded the discussion to better interpret and highlight the significance of our results. Specifically, we have added detailed analyses and implications of our findings on lines 249 to 253 and lines 286 to 289 of the revised manuscript. These additions aim to clarify how our results can impact clinical practice and set the ground for future studies.
